# What makes a great radiology review course lecture: the Ottawa radiology resident review course experience

**DOI:** 10.1186/1472-6920-14-22

**Published:** 2014-02-01

**Authors:** Lilly Cao, Matthew DF McInnes, John O Ryan

**Affiliations:** 1 Department of Medical Imaging, Ottawa Hospital Research Institute, Ottawa, Canada; 2 University of Ottawa, Ottawa, Canada

## Abstract

**Background:**

Little objective evidence exists regarding what makes a good lecture. Our purpose was to determine qualities of radiology review course lectures that are associated with positive audience evaluation.

**Methods:**

57 presentations from the Ottawa Resident Review Course (2012) were analyzed by a PGY4 radiology resident blinded to the result of audience evaluation. Objective data extracted were: slides per minute, lines of text per text slide, words per text slide, cases per minute, images per minute, images per case, number of audience laughs, number of questions posed to the audience, number of summaries, inclusion of learning objectives, ending on time, use of pre/post-test and use of special effects. Mean audience evaluation scores for each talk from daily audience evaluations (up to 60 per talk) were standardized out of 100. Correlation coefficient was calculated between continuous variables and audience evaluation scores. Student *T* test was performed on categorical variables and audience evaluation scores.

**Results:**

Strongest positive association with audience evaluation scores was for image quality (r = 0.57) and number of times the audience laughed (r = 0.3). Strongest negative association was between images per case and audience scores (r = -0.25). Talks with special effects were rated better (mean score 94.3 vs. 87.1, p < 0.001). Talks with the highest image quality were rated better (mean score 94.1 vs. 87.5, p < 0.001). Talks which contained a pre/post-test were rated better (mean score 92 vs. 87.8, p = 0.004).

**Conclusion:**

Many factors go into making a great review course lecture. At the University of Ottawa Resident Review Course, high quality images, use of special effects, use of pre/post-test and humor were most strongly associated with high audience evaluation scores. High image volume per case may be negatively associated with audience evaluation scores.

## Background

Educational presentations in Diagnostic Imaging are often crafted through experience, intuition, and based on feedback from previous lectures. Although many articles have been written about what goes into creating a great radiology lecture, as well as lectures in general, these are often not based on objective data, but are in the domain of ‘expert opinion’ [1-5]. Little is known regarding relationship of certain lecture variables (e.g. number of cases, number of slides) with lecture effectiveness.

Many traits have been associated with effective presentations although have not necessarily been objectively studied to determine if they correlate with audience evaluation. These would include lectures with clearly stated objectives, high quality images, techniques that encourage audience participation such as audience questioning, as well as strategies to motivate and entertain the listener including humor [1]. Other traits such as text slides with too many lines per slide and too many words per slide have been associated with lower quality presentations [2]. Analysis of comments received at a National Radiology Continuing Medical Education Course demonstrated that poor image quality such as images that are too dark or incompletely projected on a screen commonly resulted in negative feedback [3].

Radiology resident review courses are common and aim to prepare residents for board examinations [6-9]. Lecturing at these courses can be demanding and may require a more case-intensive style than lectures given at other diagnostic imaging courses. The University of Ottawa puts on an annual week long resident review course that aims to prepare residents for their Canadian Radiology Board Examinations [9]. This course was started in 2011 and has been attended by more than 500 individuals over 3 years. In order to continually improve course quality, the course directors gather feedback from attendees regarding each lecture.

The purpose of our study is to evaluate which radiology review course lecture variables are associated with positive audience evaluation.

## Methods

The local research ethics board waived approval for this study. Ottawa Hospital Research Ethics Board.

The 2nd Annual Ottawa Resident Review Course which took place from March 25–30, 2012 had a total of 57 presentations given by 39 separate speakers; it was attended by more than 150 people. The vast majority (>80%) of attendees were radiology residents from Canadian residency programs; the remaining attendees were a combination of residents from American radiology residency programs and radiologists practicing in Canada (Reference: personal communication with Sandra Leslie: May 2013). Video capture of the slides and simultaneous audio of the presenters was saved. PDF versions of each talk were also saved for review. Forty six of the 57 presentations had video files which could be reviewed (some speakers did not consent to recording); all 57 presentations had pdf files available. Audience evaluations of each lecture from course attendees were collected. Lectures were scored on a 1–5 scale (5 being best) and freeform comments could be made. These were standardized to a maximum score of 100 by adding all scores achieved by a given talk, multiplying by 20 and then dividing by the total number of respondents.

### Data extraction

The following objective data was collected by reviewing the recorded lectures (when available) or the lecture pdf files: use of objectives or outline, total slides per minute, number of text lines per text slide, number of words per text slide, cases per minute, images per minute, images per case, number of episodes of audience laughter per presentation, number of questions posed to the audience per presentation, number of summaries or summary slides, use of animation. ‘Total slides’ was defined as all slides contained in the presentation. ‘Text slides’ was defined as slides containing only text content and no images. Lectures were classified as either ‘didactic’ or ‘unknown case presentations’. Lecture were classified as ‘unknown case presentations’ if the majority of cases were initially presented without an associated diagnosis.

Image quality was assessed by reviewing recorded lectures and scored on a subjective 1–5 scale with 5 being best. Higher scores were awarded to talks with images that were properly cropped and possessed suitable contrast and clearly demonstrated the relevant findings. Presentations with these traits for all of the images were scored as 5, for only half were scored as 3 and for none were scored as 1. The basis for the framework of the image quality evaluation was derived from frequently cited image quality criticisms in a prior study of radiology lectures [3].

Data collection was done by (L.C.) a PGY4 radiology resident. The data collector was not blinded to the speaker identity; this was not feasible due to the fact that speakers were recognizable from the audio files.

Written comments from course evaluations were reviewed qualitatively. Commonly recurring comments were tabulated. Common occurrence was defined as occurring more than 3 times.

### Data analysis

Pearson correlation coefficients were calculated between each of the extracted variables with non-dichotomous scoring and the presentation evaluation scores by attendees.

For the variables with dichotomous scoring, a student *t*-test analysis was performed (use of objectives/outlines, presence of audience laughter, finishing on time, use of summaries during the presentation, questions posed to the audience, use of pre or post-test, unknown vs. didactic, use of special effects such as animation). Talks with slides per minute in excess of one standard deviation (SD) above the mean were compared to the remainder of the presentations. For the 55 presentations with images, lectures with cases per minute, images per minute or images per case one SD above the mean were compared to the remaining talks. Similarly, of the 44 talks on video which also contained images, those achieving image quality scores of 5 were compared to talks awarded scores of 1 to 4.

A low score group and a high score group were defined as talks awarded a standardized feedback score of less than one SD or more than one SD relative to the mean overall feedback score, respectively. Average values for many of the above described parameters were compared between the high scoring and low scoring groups using the Student *t*-test. Parameters analyzed as a proportion were defined with 0 being none of the presentations in the group possessing a characteristic, 1 being all of the presentations possessing a characteristic and values in between corresponding to the fraction of talks displaying the trait in question.

Statistical analysis was performed with Microsoft Excel (Microsoft Corporation, Redmond Washington, 2013, version 15.0.4454.1503).

## Results

The number of evaluations received for each presentation ranged from 26–60 with a mean of 47.8. Presentation scores ranged in value from 72 to 97 (out of 100). An average score of 87.8 was achieved with a standard deviation of 6.5. There were 9 presentations which achieved a score of greater than 94.3, defined as the “high scoring group”. There were 11 presentations that received a score of less than 81.3, defined as the “low scoring group”. Of the 57 presentations, 55 contained Diagnostic Imaging (DI) images, such as images from MRI, CT, US and plain radiography studies.

The commonly recurring written qualitative positive comments amongst the high scoring group presentations and the commonly encountered written negative comments amongst the low scoring group presentations are presented in Table 1.

**Table 1:** Recurring comments for HSG presentations and LSG presentations

**HSG presentations:**	**LSG presentations:**
Lots of cases	Handout and talk do not match
Beautiful cases	Inaccurate or mistakes
Good pace/speed	No differentials. No differential diagnosis slides. Too fast when discussing differentials
Clear/organized	Too slow too fast.
Labels	Too quiet. Cannot hear.
Material resembles exam	Images bad, too small, too many
	Not enough cases
	Disorganized
	Incomplete

Correlation between presentation scores and various parameters are listed in Table 2. The strongest correlation was between presentation scores and image quality scores, r = 0.57. The second strongest correlation was identified between presentation scores and the number of times the audience laughed during the talk, r = 0.3. The strongest negative correlation was between presentation scores and ‘images per case’ r = -0.25.

**Table 2:** Correlation between presentation score and various characteristics

**Parameter**	**Number of presentations**	**Correlation coefficient**
Image quality score	44	0.57
Number of times audience laughed	46	0.3
Slides per minute	57	0.1
Number summaries during talk	46	0.06
Number of questions for audience	46	0.05
Cases per minute	55	-0.0048
Text lines per text slide	57	-0.1
Images per minute	55	-0.176
Words per text slide	57	-0.2
Images per case	55	-0.25

Two presentations had no Diagnostic Imaging images.

Comparison of scores based on various characteristics is summarized in Tables 3, 4 and 5. Presentations with sophisticated special effects demonstrated an average score of 94.3 whereas the remaining presentations had an average score of 87.1 (p < 0.01) (Table 3). Presentations which contained one or both of a pretest or posttest component scored an average of 92 whereas those with neither scored an average of 87.8 (p = 0.004) (Table 3). Presentations with an image quality score of 5 received an average score of 94.1 compared to the rest of the presentations which were awarded an average score of 87.5 (p < 0.01) (Table 4). An interesting outcome was that the average score for presentations with images per case in excess of one standard deviation above the mean was 83.4 whereas for the remainder of the presentations was 88.8 (p = 0.027) (Table 5).

**Table 3:** Comparison of scores based on presence or absence of a feature

	**Present**	**Average score**	**Absent**	**Average score**	**p value**
Contains sophisticated special effects	10	94.3	34	87.1	3.83 × 10e-7
Contains at least one of either a pretest or posttest	6	92	40	87.8	0.004
Audience laughed during talk	11	91	35	87.5	0.07
Objectives or outline	30	87.1	27	88.5	0.22
Presentation was on time	30	88.9	16	87.5	0.298
Summary occurred during talk	27	88	19	88.9	0.34
Didactic lecture vs. unknown cases	28 (didactic)	88.6	16 (unknown)	88.7	0.95
Questions for audience	8	88.3	38	88.4	0.95

46 talks with video, 44 talks with DI images.

44 talks with images.

**Table 4:** Comparison of scores based on reviewer scores from video review

**Parameter**	**Talks with score of 5**	**Average score**	**Talks with score less than 5**	**Average score**	**p value**
Image quality score (1-5)	8	94.1	36	87.5	9.34 × 10e-6

46 talks with video, 44 of these had DI Images.

**Table 5:** Comparison of presentation scores based on a parameter value

**Parameter**	**Average**	**One SD above mean**	**Number of presentations one SD above mean**	**Average score**	**Number of remaining presentations**	**Average score**	**p value**
Images per case	3.9	6	8	83.4	47	88.8	0.027
Images per minute	3.4	5.2	6	84.5	49	88.4	0.16
Slides per minute	2.69	3.72	5	89.8	52	87.6	0.47
Cases per minute	1	1.55	8	86.7	47	88.2	0.54

Tables 6 and 7 summarizes the comparison of the high score group versus the low score group across a variety of parameters. Between the high score group and low score group, average image quality scores revealed a difference of 4.4 vs. 3.6 (p = 0.006) while the proportion of talks with and without sophisticated special effects demonstrated a difference of 0.625 vs. 0 (p = 0.006).

**Table 6:** Comparison of scores between HSG and LSG based on video review

**Parameter**	**HSG average**	**LSG average**	**p value**
Image quality score (1-5)	4.375	3.57	0.0056
Proportion containing sophisticated special effects	0.625	0	0.0056
Number of times audience laughed	1.625	0	0.051
Proportion containing pretest or posttest	0.125	0	0.175
Proportion that finished on time	0.875	0.75	0.28
Number of summaries	0.75	1	0.51
Number of questions for audience	1.75	1.25	0.75
Proportion that were didactic lectures (with the rest being unknown type lectures)	0.5	0.5	N/A

All HSG scores include 8 presentations. All LSG scores include 8 presentations except Image quality score, Proportion containing Sophisticated Special Effects and Proportion containing any Special Effects which each contain 7 as one talk did not have Diagnostic Imaging images.

**Table 7:** Comparison of scores between all HSG and LSG presentations

**Parameter**	**HSG average**	**LSG average**	**p value**
Words per text slide	27.4	32	0.39
Slides per minute	2.9	2.6	0.47
Text lines per text slide	6.3	6.7	0.61
Images per case	4	4.3	0.76
Cases per minute	1.01	0.97	0.85
Images per minute	3.41	3.55	0.86
Proportion with objectives or outline	0.556	0.636	0.88

9 talks in HSG group and 11 talks in LSG group.

## Discussion

Consistent with previous reports [2], our results indicate that high quality images that were properly cropped, well projected, possessed suitable contrast and clearly demonstrated the relevant findings was most strongly associated with higher attendee evaluation scores. The second most strongly correlated variable with higher scores was the “number of times the audience laughed” suggesting that humor may be influential in achieving more positive feedback. This would support the role of entertainment in maintaining audience interest and attention to establish an environment conducive to learning [1]. It is interesting to note that this metric only achieved near statistical significance when comparing lectures with and without audience laughter.

It should be pointed out that the correlations in our study were not particularly strong; the strongest for image quality scores at r = 0.57 and the remainder less than 0.5, suggesting that determinants of a high quality Diagnostic Imaging lecture are likely multifactorial.

The use of special effects was also strongly associated with higher scores. This would include the use of clear annotations pointing to the appropriate findings as well as effective use of animation including builds and transitioning between slides [2].

Using a pretest or posttest to interactively focus the audience’s attention on salient points of a lecture was also found to be associated with a higher score. This emphasizes the central role that audience interaction and participation plays in the dynamic learning process [1]. However, simply having informal questions posed to the audience was not associated with higher scores.

It is interesting that many factors which might intuitively be expected to be associated with higher evaluation scores were not confirmed in our study. This would include having stated objectives or summarizing material during the lecture both of which have been felt to enhance speaker effectiveness [1]. Not finishing on time was previously documented to be a common source of negative audience feedback [3] although this was not confirmed to be statistically significant in our results. As well, it has long been thought that text slides with too many words per slide or too many text lines per slide were ineffective [2,3] although in our study this revealed only fairly weak negative correlation with audience evaluation scores.

Despite radiology being an image based specialty, a higher number of images were not associated with higher audience evaluation scores. In fact, having more images per case was the strongest negative correlation in our study although is not incompatible with previous reports [2]. This would suggest that image quality is much more important than image quantity. This might be explained by the fact that more images may result in a disorganized or rushed lecture perhaps with lower quality images which may not effectively convey the presenter’s message. It has been suggested that images should not be overused or include simply to impress the audience. Superfluous images can be avoided by eliminating anything that does not assist in attainment of the original lecture objectives [2].

An additional interesting finding is that the HSG and LSG (Tables 3 and 6) contain the same proportion of didactic vs. case-based lectures indicating that lecture style alone is not a determinant of success or failure.

A weakness of our study includes the fact that not all presentations had video files that were available to be reviewed. The conference that we studied was targeted to a specific audience of resident review course attendees which may limit the applicability to other courses in radiology and other specialties. Similar studies at different CME conferences may help identify whether these patterns endure at courses with other themes. Additional limitations include varied number of audience evaluations (ranging from 20-50% of attendees), and the fact that a single individual extracted all of the objective data.

The comments from attendees point to some possible areas for further research such as organization, pace of talk, volume of speaker and accuracy of slides.

## Conclusion

This study identifies that there are many determinants of high quality Diagnostic Imaging review course lectures. The factors that most strongly contribute to lecture success are: high quality images; use of fewer images per case; use of special effects which clearly and precisely convey imaging findings or clarify difficult concepts; use of pretest/posttest tools and perhaps most importantly—a sprinkling of humor. These findings can assist in optimizing lecture preparation and guide further research.

## Competing interests

The authors declare that they have no competing interests.

## Authors’ contributions

LC performed data extraction, drafted the manuscript and approved the final version of the manuscript. MM helped with study design, manuscript editing and approved the final version of the manuscript. JR helped with study design, manuscript editing and approved the final version of the manuscript.

## Pre-publication history

The pre-publication history for this paper can be accessed here:

http://www.biomedcentral.com/1472-6920/14/22/prepub
